# Ethyl Acetate Abatement on Copper Catalysts Supported on Ceria Doped with Rare Earth Oxides

**DOI:** 10.3390/molecules21050644

**Published:** 2016-05-17

**Authors:** Sónia Alexandra Correia Carabineiro, Michalis Konsolakis, George Emmanouil-Nontas Marnellos, Muhammad Faizan Asad, Olívia Salomé Gonçalves Pinto Soares, Pedro Bandeira Tavares, Manuel Fernando Ribeiro Pereira, José Joaquim de Melo Órfão, José Luís Figueiredo

**Affiliations:** 1 Laboratório de Catálise e Materiais (LCM), Laboratório Associado LSRE-LCM, Faculdade de Engenharia, Universidade do Porto, 4200-465 Porto, Portugal; faizan_asad@hotmail.com (M.F.A.); salome.soares@fe.up.pt (O.S.G.P.S.); fpereira@fe.up.pt (M.F.R.P.); jjmo@fe.up.pt (J.J.M.Ó.); jlfig@fe.up.pt (J.L.F.); 2 School of Production Engineering and Management, Technical University of Crete, 73100 Chania, Greece; mkonsol@science.tuc.gr; 3 Department of Mechanical Engineering, University of Western Macedonia, GR-50100 Kozani, Greece; gmarnellos@uowm.gr; 4 Chemical Process & Energy Resources Institute, Centre for Research & Technology Hellas, GR-57001 Thessaloniki, Greece; 5 CQVR—Centro de Química–Vila Real, Departamento de Química, Universidade de Trás-os-Montes e Alto Douro, 5001-911 Vila Real, Portugal; ptavares@utad.pt

**Keywords:** ethyl acetate oxidation, volatile organic compounds (VOCs), copper (Cu)-based catalysts, mixed oxides, rare earth oxides

## Abstract

Different lanthanide (Ln)-doped cerium oxides (Ce_0.5_Ln_0.5_O_1.75_, where Ln: Gd, La, Pr, Nd, Sm) were loaded with Cu (20 wt. %) and used as catalysts for the oxidation of ethyl acetate (EtOAc), a common volatile organic compound (VOC). For comparison, both Cu-free (Ce-Ln) and supported Cu (Cu/Ce-Ln) samples were characterized by N_2_ adsorption at −196 °C, scanning/transmission electron microscopy, energy-dispersive X-ray spectroscopy, X-ray diffraction, X-ray photoelectron spectroscopy and temperature programmed reduction in H_2_. The following activity sequence, in terms of EtOAc conversion, was found for bare supports: CeO_2_ ≈ Ce_0.5_Pr_0.5_O_1.75_ > Ce_0.5_Sm_0.5_O_1.75_ > Ce_0.5_Gd_0.5_O_1.75_ > Ce_0.5_Nd_0.5_O_1.75_ > Ce_0.5_La_0.5_O_1.75_. Cu addition improved the catalytic performance, without affecting the activity order. The best catalytic performance was obtained for Cu/CeO_2_ and Cu/Ce_0.5_Pr_0.5_O_1.75_ samples, both achieving complete EtOAc conversion below *ca.* 290 °C. A strong correlation was revealed between the catalytic performance and the redox properties of the samples, in terms of reducibility and lattice oxygen availability. Νo particular correlation between the VOC oxidation performance and textural characteristics was found. The obtained results can be explained in terms of a Mars-van Krevelen type redox mechanism involving the participation of weakly bound (easily reduced) lattice oxygen and its consequent replenishment by gas phase oxygen.

## 1. Introduction

Volatile organic compounds (VOCs) are environmental pollutants regarded as precursors for the formation of tropospheric ozone (a greenhouse gas) and photochemical smog. They originate from loading operations, motor vehicles, solvent cleaning, printing and painting operations, refineries and fuel storage installations [1]. Common VOCs are ethyl acetate (EtOAc), toluene, benzene, ethanol and acetone [2].

Catalytic oxidation is a promising VOC abatement technology, requiring lower temperatures (around 250–500 °C) than conventional thermal oxidation processes (which operate at 650–1100 °C), and thus less energy requirements, while at the same time being associated with lower NO_x_ emissions. Hence, it can be considered as an environmentally-friendly and cost-effective technology [3,4].

Several catalysts have been tested in VOC oxidation, including titania [3], alumina [5], zirconia [6], zeolites [7] and carbon-based materials [4]. Noble metal (NM)-based catalysts are very efficient, stable and exhibit high activity at low temperatures. Platinum and palladium are often used [8,9,10], but gold has also been recently investigated [11,12,13,14,15,16,17]. The development of NM-free catalytic systems is of growing interest, taking into account their lower cost. Common metal oxide catalysts are: manganese oxides [8,15,18], copper oxide [4,7,19], nickel oxide [7,14,19], iron oxide [19,20], cobalt oxide [19,20,21,22], among others [20]. Moreover, mixed oxides [14,23,24,25,26,27,28,29], like perovskites [30,31,32] and cryptomelane-type materials [33,34,35] have been widely employed for VOCs abatement. In particular, Cu-based oxides [25,36], such as CuO-CeO_2_ [24,26,37,38,39,40] and CuO-Co_3_O_4_ [41,42,43] demonstrate superior oxidation performance. This is mainly attributed to their enhanced reducibility [38,39,40], which is considered as one of the most important factors influencing VOC oxidation activity [15,33,35,44,45,46].

Recently, several strategies have been used to modify the oxygen storage capacity (and consequently the reducibility) of bare ceria [36]. Doping CeO_2_ with aliovalent metal ions can affect its redox properties and, therefore, its catalytic efficiency [15,24,26,36,40,42,46,47,48]. For example, an enhanced reducibility and catalytic performance towards the NO reduction by CO was obtained by doping Cu/Al_2_O_3_ catalysts with Ce/Zr mixed oxides [49]. In a similar manner, Cu-Ce_0.75_Zr_0.25_/ZSM-5 composites exhibited high catalytic activity for EtOAc oxidation, with complete conversion at *ca.* 270 °C [50]. Their superior performance was mainly ascribed to the synergistic effect between Cu–Ce–Zr towards an improved oxygen mobility and enhanced reducibility [50]. Recently, Ce_0.5_Pr_0.5_O_δ_ mixed oxides demonstrated remarkable behavior in the catalytic combustion of dichloroethane [51].

The aim of this work is to explore the impact of ceria doping by lanthanide (Ln) elements, *i.e.*, Gd, La, Nd, Pr and Sm, on the physicochemical properties and VOCs oxidation performance of Cu/Ce_0.5_Ln_0.5_O_1.75_ catalysts. In order to assess the effect of Ln- and Cu-doping on ceria, the solid state properties and the catalytic performance of both Ce_0.5_Ln_0.5_O_1.75_ (Ce-Ln) and Cu/Ce_0.5_Ln_0.5_0_1.75_ (Cu/Ce-Ln) oxides were comparatively examined. The textural, structural, morphological, redox and surface properties of all samples were evaluated by N_2_ adsorption at −196 °C, X-ray diffraction (XRD), scanning/transmission electron microscopy (SEM/TEM) and energy-dispersive X-ray spectroscopy (EDS), temperature programmed reduction in H_2_ (H_2_-TPR) and X-ray photoelectron spectroscopy (XPS), in order to reveal possible structure-activity relationships.

## 2. Results

### 2.1. Textural Characterization

The main textural (BET surface area, total pore volume) and structural (phases detected and their crystallite sizes) properties of both Ce-Ln and Cu/Ce-Ln samples are presented in Table 1. Bare ceria shows optimum textural characteristics, in terms of surface area (72 m^2^/g) and pore volume (0.27 cm^3^/g). Ln-doping resulted in a significant decrease of BET area, which is further intensified upon Cu addition (20 wt. %). Amongst the Ce-Ln samples, Ce-Gd showed the lowest BET area (5 m^2^/g), while Ce-Nd had the highest (35 m^2^/g). Intermediate values were obtained for Ce-La (25 m^2^/g), Ce-Sm (21 m^2^/g) and Ce-Pr (22 m^2^/g). The same trend was followed for Cu-doped samples, demonstrating, however, slightly lower values in comparison to Cu-free samples. These findings clearly imply the blockage of ceria pores by rare earth oxides during the calcination procedure, which however, is strongly dependent on the nature of the lanthanide element. This can be, most probably, attributed to the different structural features (phases detected and their crystallite sizes) of Ce-Ln and Cu/Ce-Ln samples, as discussed below. It is interesting to note that, for most samples, the incorporation of Cu only slightly affects the surface area. However for CeO_2_ and Ce-Pr materials, the initial BET surface areas are significantly decreased upon Cu loading. This might be due to close interactions between copper and the support, which may lead to strong metal-support interactions.

### 2.2. XRD

**Figure 1:**
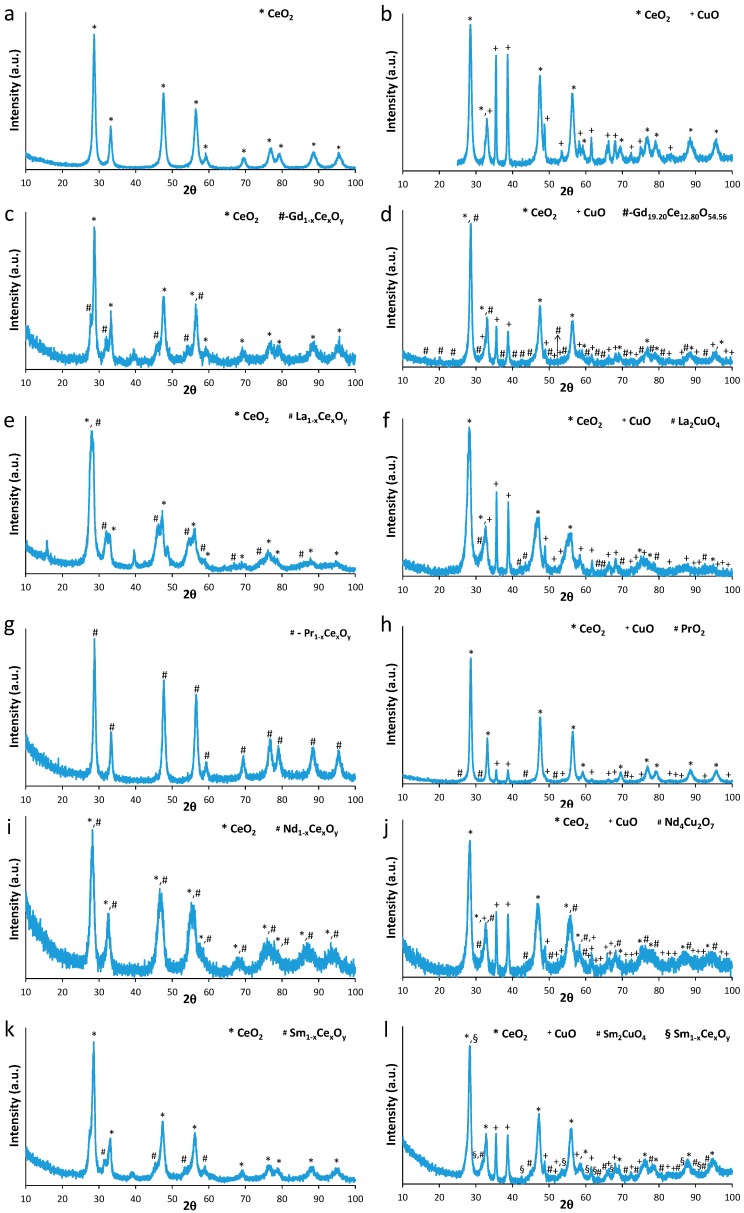
XRD patterns of CeO_2_ (**a**); Cu/CeO_2_ (**b**); Ce-Gd (**c**); Cu/Ce-Gd (**d**); Ce-La (**e**); Cu/Ce-La (**f**); Ce-Pr (**g**); Cu/Ce-Pr (**h**); Ce-Nd (**i**); Cu/Ce-Nd (**j**); Ce-Sm (**k**) and Cu/Ce-Sm (**l**).

The structural characteristics of Ce-Ln and Cu/Ce-Ln materials were obtained by XRD analysis (Figure 1 and Table 1). In Ce-Ln oxides, the cerianite phase is present in CeO_2_, Ce-Gd, Ce-La and Ce-Sm samples with crystallites sizes between 15.9 and 18 nm. It has a cubic $Fm\bar{3}m$ (225) structure with lattice parameter a = 5.41 Å for pure ceria. Rare earth (RE)-doping (La, Pr, N, Sm or Gd) in a molar amount equivalent to Ce, resulted in the formation of different phases depending on Ln nature: (a) for Ln = Pr or Nd, *i.e.*, for the lanthanide elements closer to Ce in the Periodic Table, the formation of a solid solution of the Ln_1−x_Ce_x_O_y_ type was found, with a cubic structure and lattice parameters of 5.43 Å for Pr_1-x_Ce_x_O_y_ and of 5.49 Å for Nd_1−x_Ce_x_O_y_; (b) for Ln = Sm, Gd and La the formation of two cubic phases, one similar to CeO_2_, with lattices of 5.44 Å (Ce-Sm) and 5.43 Å (Ce-Gd) and another of Ln_1-x_Ce_x_O_y_ type with lattice parameters of 5.64 Å for Sm_1−x_Ce_x_O_y_, 5.62 Å for Gd_1−x_Ce_x_O_y_ and 5.56 Å for La_1-x_Ce_x_O_y_. It seems obvious that there is a solid solution limitation in the cases of Ce-Sm, Ce-Gd and Ce-La oxides. In fact, the amount of the Ln_1−x_Ce_x_O_y_ solid solution increases in the following order: Ce-Gd (18.3%) < Ce-Sm (22.2%) < Ce-La (64.8%) < Ce-Nd (100%) = Ce-Pr (100%).

Upon Cu addition, the crystallite size of cerianite (present in Cu/CeO_2_, Cu/Ce-Gd, and Cu/Ce-Sm materials) showed some changes, due to the additional calcination of Ce-Ln samples after Cu loading. Independently of the support nature, copper was always crystallized in the monoclinic structure as CuO-tenorite. Its crystallite size varied between 10 and 12 nm for Cu/Ce-Gd, Cu/Ce-La, Cu/Ce-Nd and Cu/Ce-Sm samples. It was larger for Cu/Ce-Pr (48 nm) and for Cu/CeO_2_ (70 nm). In the Cu/Ce-La sample, a La_2_CuO_4_ cubic phase was also detected (8.6%), while for Ce/Ce-Sm, a Sm_2_CuO_4_ tetragonal phase was present (9.2%).

### 2.3. H_2_-TPR

**Figure 2:**
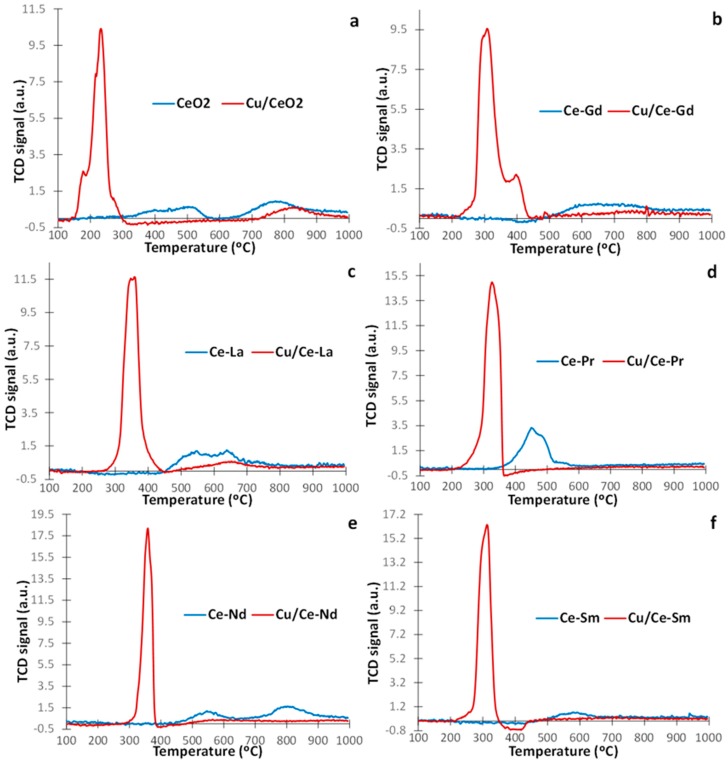
TPR profiles of samples with and without Cu: CeO_2_ (**a**); Ce-Gd (**b**); Ce-La (**c**); Ce-Pr (**d**); Ce-Nd (**e**) and Ce-Sm (**f**).

TPR studies were carried out to gain insight into the impact of the support on the redox properties of the samples. Figure 2 depicts the TPR profiles for both Ce-Ln and Cu/Ce-Ln samples. Bare CeO_2_ (Figure 2a) shows two peaks centred at *ca*. 500 and 780 °C, attributed to the reduction of surface and bulk oxygen, respectively [36,48,52,53,54,55,56,57,58,59].

It has been well established that CuO shows two reduction peaks at *ca*. 300 °C, which correspond to the stepwise reduction of CuO to metallic Cu (Cu^2+^ → Cu^+^ → Cu°) [16,17,19,60]. However, metal (Cu) incorporation into the lattice of metal oxides (Ce-Ln) notably facilitates the surface-shell reduction, shifting the TPR peaks to lower temperatures (Figure 2). A similar behaviour was observed with the incorporation of gold on ceria [15,57,58,59]. Therefore, the overlapping peaks in the temperature range of 200–450 °C for Cu/Ce-Ln samples (Figure 2 and Table 1) can be assigned to the reduction of copper oxides along with the facilitated reduction of surface oxygen of lanthanide oxides. The small peaks at higher temperatures (above 500 °C) are due to bulk oxygen reduction of lanthanide oxides [36,48,52,53,54,55,56,57,58,59,61,62]. Addition of Cu does not seem to have an influence on the higher temperature peaks, as also reported for gold addition to ceria [15,57,58,59]. In fact, these peaks are slightly decreased in the presence of Cu.

In terms of onset temperatures, Cu/CeO_2_ presents the lowest value (139 °C), followed by Cu/Ce-Pr (207 °C), Cu/Ce-Gd (233 °C) and Cu/Ce-Sm (255 °C), while Cu/Ce-Nd and Cu/Ce-La present the highest (around 280 °C), as seen in Figure 2 and Table 1. In relation to Cu-free samples, CeO_2_ has the lowest value (164 °C), followed by Ce-Pr (312 °C), Ce-Nd (390 °C), Ce-Gd (420 °C), Ce-La (424 °C) and Ce-Sm (429 °C).

### 2.4. SEM/EDS

**Figure 3:**
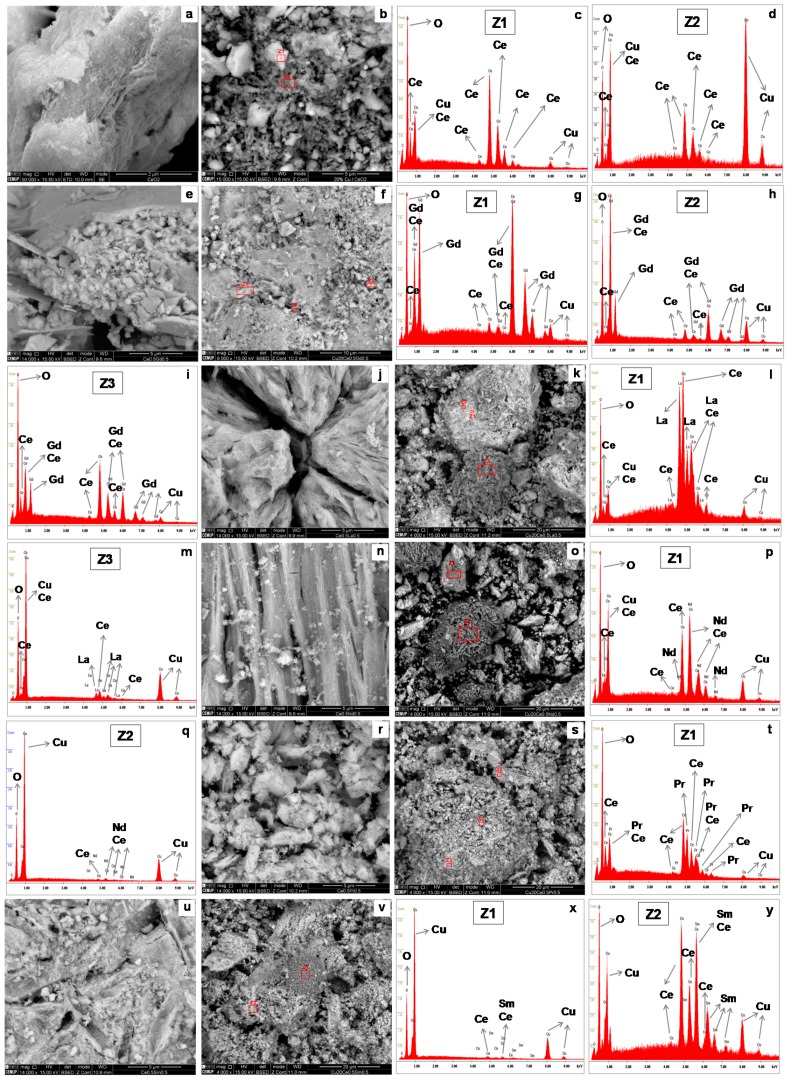
SEM images of CeO_2_ (**a**); Cu/CeO_2_ (**b**) and respective EDS spectra of zones marked as Z1 (**c**) and Z2 (**d**); Ce-Gd (**e**); Cu/Ce-Gd (**f**) and respective EDS spectra of zones marked as Z1 (**g**), Z2 (**h**) and Z3 (**i**); Ce-La (**j**); Cu/Ce-La (**k**) and respective EDS spectra of zones marked as Z1 (**l**) and Z3 (**m**); Ce-Nd (**n**); Cu/Ce-Nd (**o**) and respective EDS spectra of zones marked as Z1 (**p**) and Z2 (**q**); Ce-Pr (**r**); Cu/Ce-Pr (**s**) and respective EDS spectra of zone marked as Z1 (**t**); Ce-Sm (**u**); Cu/Ce-Sm (**v**) and respective EDS spectra of zones marked as Z1 (**x**) and Z2 (**y**).

The morphological characteristics of Ce-Ln and Cu/Ce-Ln samples were investigated by SEM/EDS (Figure 3). Figure 3a corresponds to the CeO_2_ material, which shows a homogenous appearance. Addition of Cu to CeO_2_ (Figure 3b) results in a heterogeneous mixture of “lighter” areas (marked as Z1, as seen in the EDS spectrum of Figure 3c) and “darker” zones (marked as Z2) that are Cu-rich, as shown in Figure 3d. Ce-Gd (Figure 3e) and Cu/Ce-Gd (Figure 3f) are similar and show some parts resembling “veils” (the area marked as Z1 is an example) that are Gd-rich, as seen in the EDS spectrum of Figure 3g, “darker” zones in smaller amounts (marked as Z2) that are Cu-rich, as shown in Figure 3h, and “lighter” areas (marked as Z3) that are Ce-rich, as depicted in Figure 3i. Figure 3j depicts a closer detail of the Ce-La material, showing a compact structure with some “cracks”. Figure 3k depicts the Cu/Ce-La sample, where three different types of areas can be distinguished: lighter (Z1), intermediate colour (Z2) and darker (Z3). EDS showed that Z1 and Z2 are very similar in composition (only Z1 zone is shown in Figure 3l for simplicity), being La-rich, while Z3 has more Cu (Figure 3m). Figure 3n shows a closer detail of the Ce-Nd sample that exhibits a “bamboo”-like structure and some lighter small portions similar to “cotton”. Similar distinct areas are observed for Cu/Ce-Nd (Figure 3o): lighter portions with a “rocky” appearance (Ce- and Nd- rich, as shown in Figure 3p) and darker, rougher agglomerates (Cu-rich, as seen in Figure 3q). Figure 3r shows a closer detail of the Ce-Sm sample depicting some homogenous agglomerates. On the Cu/Ce-Pr sample (Figure 3s): lighter agglomerates (Z1), darker parts (Z2) and some pieces with a “rocky” smoother appearance (Z3) are observed. Their composition is similar, as shown in Figure 3t (EDS of zone Z1). Figure 3u shows a closer detail of a lighter part of the Ce-Sm sample, which is similar to what is observed for Cu/Ce-Sm (Figure 3v). The darker parts are Cu-rich (Figure 3x) and the lighter areas are Ce and Sm-rich (Figure 3y).

### 2.5. TEM

**Figure 4:**
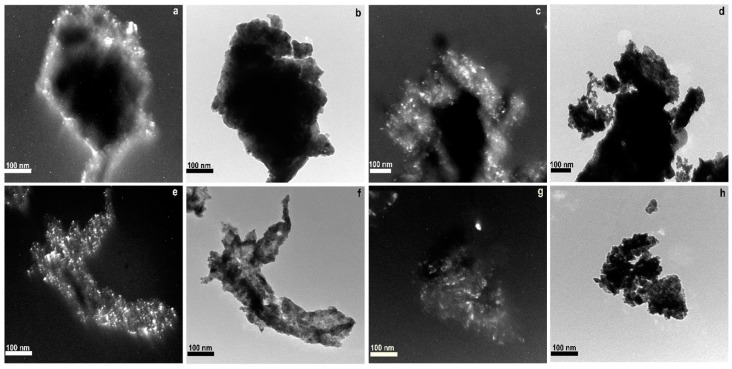
TEM images of Cu/Ce_-_Ln (Ln: Gd, La, Pr, Nd, Sm) samples. Cu/Ce-Gd: dark-field (**a**) and bright field (**b**) images; Cu/Ce-La: dark-field (**c**) and bright field (**d**) images; Cu/Ce-Pr dark-field (**e**) and bright field (**f**) images and Cu/Ce-Nd dark-field (**g**) and bright field (**h**) images.

Samples with Cu were also imaged by TEM (Figure 4). Figure 4a,c,e,g show TEM dark-field images that can be used to demonstrate that particles are crystalline. The bright field TEM images of the same portions of sample (Figure 4b,d,f,h) show darker areas. Particle size measurements in these figures correlate well with the crystallite sizes obtained from XRD (Table 1). For example in Cu/Ce-La two different particle sizes distribution are detected in TEM figures: (i) a large number of small particles, between 6 and 28 nm, corresponding to the CuO and La_1-x_Ce_x_O_y_ phases; (ii) a small number of large particles between 36 and 50 nm corresponding to the La_2_CuO_4_ phase.

### 2.6. XPS

**Figure 5:**
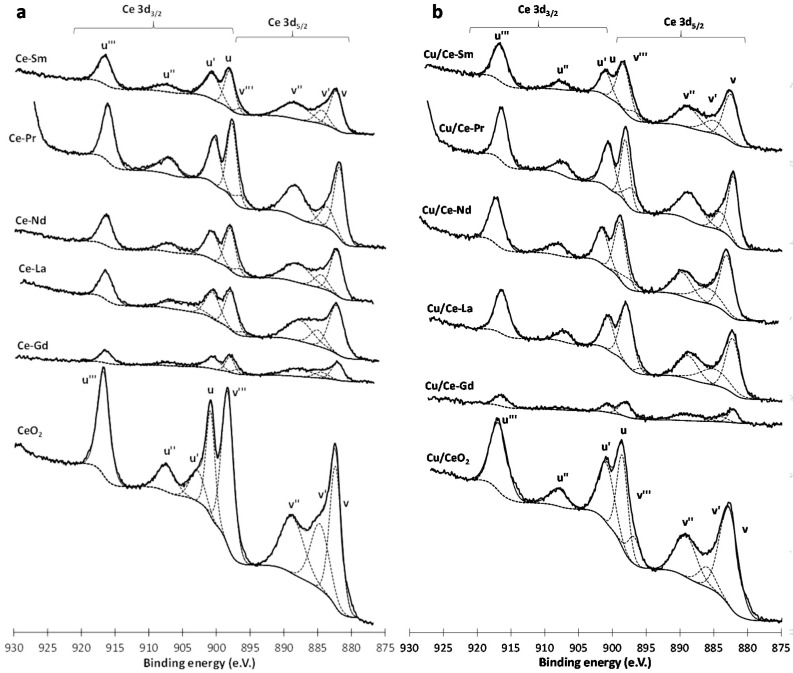
XPS Ce 3d spectra of Ce-Ln (**a**) and Cu/Ce-Ln (**b**) samples (Ln: Gd, La, Pr, Nd, Sm).

Surface analysis was carried out by XPS on both Ce-Ln and Cu/Ce-Ln samples to assess the impact of Cu- and/or Ln-doping on the surface composition and elemental chemical states. Figure 5 shows the spectra of Ce 3d for the Ce-Ln (left) and Cu/Ce-Ln (right) samples. Spectra fitting by mixed Gaussian-Lorentzian functions reveals eight peaks corresponding to Ce3d_5/2_ (v) and Ce3d_3/2_ (u) contributions. The main features at ~882 (v), ~889 (v’’), ~898 (v’’’), ~901 (u), ~907 (u’’) and ~917 eV (u’’’) are assigned to Ce^4+^, whereas those at ~896 (v’) and 903 eV (u’) to Ce^3+^ [63,64]. These doublets correspond to a mixture of the Ce3d^9^O2p^5^Ce4f^2^ and Ce3d^9^O2p^6^Ce4f^1^ final states. The concentration of Ce^3+^ in the ceria layers can be determined from the ratio: Ce^3+^/(Ce^4+^ + Ce^3+^), where Ce^3+^ and Ce^4+^ represent the sums of the integrated XPS peak areas related to Ce^3+^ and Ce^4+^ signals, respectively (Table 2). It can be seen that the relative concentration of Ce^3+^ ions is not significantly changed upon lanthanide and/or copper addition to ceria, varying from 17.82% (CeO_2_) to 23.53% (Cu/Ce-La). This is in agreement with previous studies concerning the impact of Pr-doping on CeO_2_ samples [65].

**Figure 6:**
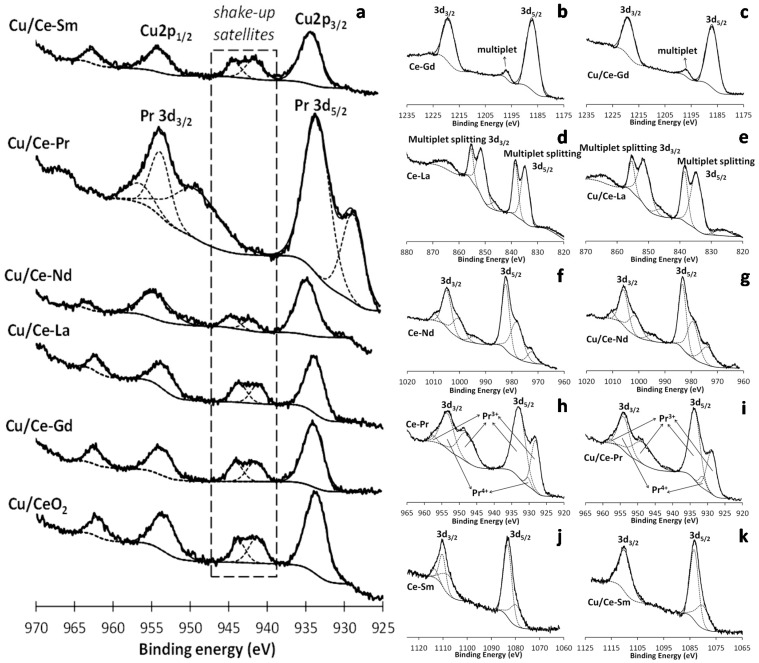
XPS Cu 2p spectra of the Cu/Ce-Ln samples, where Ln: Gd, La, Pr, Nd, Sm (**a**); Gd 3d XPS spectra of Ce-Gd (**b**) and Cu/Ce-Gd (**c**); La 3d XPS spectra of Ce-La (**d**) and Cu/Ce-La (**e**); Nd 3d XPS spectra of Ce-Nd (**f**) and Cu/Ce-Nd (**g**); Pr 3d XPS spectra of Ce-Pr (**h**) and Cu/Ce-Pr (**i**); and Sm 3d XPS spectra of Ce-Sm (**j**) and Cu/Ce-Sm (**k**).

Figure 6a shows the Cu 2p XPS spectra of the Cu/Ce-Ln samples. All spectra are characterized by two main peaks, Cu 2p_1/2_ (~954 eV) and Cu 2p_3/2_ (~934 eV), along with shake-up satellite peaks between those two. The Cu2p_3/2_ peak at ~934 eV, in combination with shake-up peaks, is typical of Cu^2+^ [47,66,67,68,69,70]. On the other hand, lower binding energies at 932–933 eV along with the absence of sattelites indicate more reduced copper species, mainly Cu_2_O [36]. According to relevant studies on XPS interpretation of Cu 2p spectra [71,72] the surface Cu^+^/Cu^2+^ ratio, can be accurately obtained by estimating the ratio of the main peak/shake-up peak areas (A1/B) for a 100% pure Cu^2+^ sample (CuO). Then, the relative concentrations of Cu^+^ and Cu^2+^ species present on the surface can be obtained by the following equations:

%Cu^+^ = (A − (A1/B)B)/(A + B) × 100 (1)

%Cu^2+^ = B(1 + (A1/B))/(A + B) × 100 (2)

where B is the area of the shake-up peak and A is the area of the main Cu 2p_3/2_ peak (Figure 6a). The qualitative analysis of the pure CuO sample gave a A1/B value of 1.89 in a previous work [73], in perfect agreement with what was found by other authors [72]. Therefore, the %Cu^+^ can be estimated in the Cu-containing samples (Table 1). Cu/Ce-Sm and Cu/Ce-La showed very low values (below 1%). Intermediate values were found for Cu/Ce-Gd (6.41%) and Cu/CeO_2_ (13.20%) samples. The highest value (29.20%) was recorded for the Cu/Ce-Nd sample. It can be seen that most Cu species in Cu/Ce-Ln samples are present as Cu^2+^ in a CuO-like phase, as shown by XRD (Table 1). These findings are in agreement with the pre-oxidation pretreatment followed in all samples before characterization studies. Moreover, previous studies over Cu catalysts supported on single or mixed REOs-based carriers indicate the predominance of Cu^2+^ ions in pre-oxidized samples [36,68]. For the Ce-Pr sample, there is a superimposition with the Pr 3d peak , preventing the calculation of the Cu^+^ species.

The core level spectra of Lanthanide elements (Ln: Gd, La, Nd, Pr, Sm) for both Ce-Ln and Cu/Ce-Ln oxides are presented in Figure 6b–k. The Gd 3d spectra of Gd oxide (Figure 6b,c) consist of a spin orbit split doublet of peaks 3d_3/2_ and 3d_5/2_, found at ~1218 and ~1187 eV, respectively [74,75,76]. The small peak at ~1196 eV is a multiplet. Therefore we can state that Gd is predominantly in the Gd^3+^ state. It is worth to point out that there is some contribution of the C KLL Auger line (expected at ~1225 eV [77]) to the Gd 3d_3/2_ peak.

The La 3d region (Figure 6d,e) is characterized by multiplet 3d_5/2_ and 3d_3/2_ splitting, due to spin-orbit interactions in addition to electron transfer from oxygen ligands to La 4f state [78]. La 3d spectra are characterized by a main 3d_5/2_ line at 834 eV and a 3d_5/2_ -3d_3/2_ spin-orbit splitting of ~17 eV, typical of La^3+^ species [78].

Nd 3d spectra (Figure 6f,g) indicate the formation of a main spin-orbit doublet, corresponding to 3d_3/2_ and 3d_5/2_ peaks at ~1004 and ~982 eV, revealing the presence of Nd^3+^ [79]. The appearance of three satellites (six peaks due to spin-orbit splitting) has been ascribed to the different final states, where the XPS core hole is screened by f electrons [80]. The superimposition of Nd 3d with O KLL Auger line (which has 3 peaks at ~978, ~999 and ~1014 eV [77]) should also be mentioned.

In the case of Pr-doped samples (Figure 6h,i), the peaks at ~932 eV (3d_5/2_) and ~952 eV (3d_3/2_) are usually assigned to Pr^4+^ species, whereas the peak pairs at 927/933 and 948/953 eV can be assigned to Pr^3+^, implying a multi-valence state of Pr in Ce-Pr mixed oxides [81]. However, it should be noted that Pr 3d peaks cannot be accurately resolved due to overlapping between Cu 2p and Pr 3d regions.

Regarding Sm-doped samples (Figure 6j,k), a major band at ~1082 eV is observed, which can be attributed to Sm^3+^ ions [26]. A broad shoulder in the low energy region is also observed, which points to the contribution of divalent (Sm^2+^) state. These findings are in agreement with the XRD results, which indicate the formation of Sm_2_CuO_4_ and Sm_1-x_Ce_x_O_1.7_ phases. Moreover, relevant literature studies have revealed the multi-valence behaviour of Sm in several intermetallic compounds [82,83].

**Figure 7:**
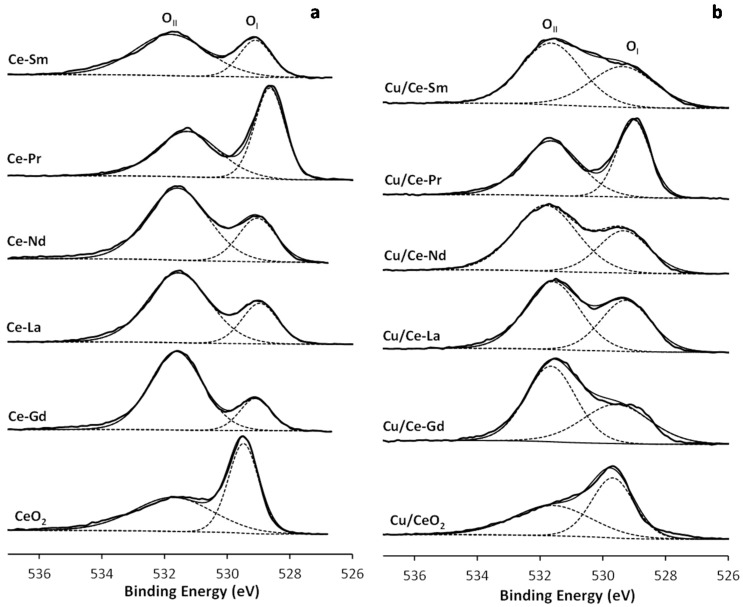
XPS O 1s spectra of the Ce-Ln (**a**) and Cu/Ce-Ln (**b**) samples, Ln: Gd, La, Pr, Nd, Sm.

Figure 7 depicts the O 1s XPS spectra, where two peaks (O_I_ and O_II_) are clearly resolved. The low binding energy peak (O_I_) at 529-530 eV originates from O^2−^ ions in the lattice, whereas the high energy peak (O_II_) at 531–533 eV may be assigned to low coordination surface oxygen species as well as to surface oxygen defects [46,84]. Hydroxyl or carbonate species can also contribute to the O_II_ peak. The relative concentration of O_I_ peaks along with O_I_/O_II_ ratios are presented in Table 2. It is evident that bare CeO_2_ and the Ce-Pr mixed oxide have the highest O_I_/O_II_ ratios (~1.0 and 0.92, respectively), followed by Ce-Sm (0.40), Ce-Nd (0.37), Ce-La (0.34) and Ce-Gd (0.25). The same trend was obtained for Cu containing samples, implying the key role of support composition on lattice oxygen. In general, CeO_2_ and Ce-Pr oxides with or without Cu exhibited very high O_I_/O_II_ ratios, varying between 0.8 and 1.0. These differences in the amount of surface oxygen species are expected to affect the redox type mechanism involved in VOCs oxidation, as will be discussed below.

The surface composition of Ce-Ln and Cu/Ce-Ln samples, in terms of Cu/Ce+Ln, Ce/Ce+Ln and Ln/Ce+Ln XPS atomic ratios, is summarized in Table 2. The corresponding nominal ratios, based on 20 wt. % Cu/Ce_0.5_Ln_0.5_O_1.75_ chemical formula, are ~0.70, 0.50 and 0.50, respectively. It is evident that the Cu/Ce+Ln atomic ratio is always (except for Cu/Ce-Pr sample) lower than the nominal one (~0.70), implying the impoverishment of catalyst surface in Cu species. In a similar manner, it has been found that the surface concentration of various transition metals supported on ceria-based composites can be remarkably decreased due to metal incorporation into the support [85,86]. On the other hand, the Ln/Ce+Ln ratio is, in general, higher than the nominal value (0.5) implying a preferential localization of lanthanide elements on the outer surface. In this regard, the enrichment of Pd/Ce-Sm catalyst surface in Sm species has been reported by other authors [87]. However this is not the case for Cu/Ce-Pr, where Ce is prevailing over Pr on the outer surface.

### 2.7. Catalytic Oxidation of EtOAc

**Figure 8:**
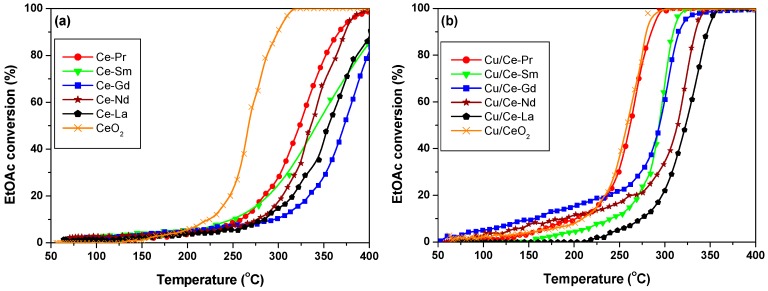
EtOAc conversion over Ce-Ln (**a**); and Cu/Ce-Ln (**b**) samples (Ln: Gd, La, Pr, Nd, Sm). Reaction conditions: [VOC]_in_ = 1000 mg_Carbon_/m^3^, GHSV = 60,000 h^−^^1^. The error for catalytic experiments is ±2 °C.

Figure 8 shows the catalytic behaviour of Ce-Ln (a) and Cu/Ce-Ln (b) oxides for EtOAc oxidation. Regarding Cu-free samples (Figure 8a), CeO_2_ is the best catalyst, achieving full conversion at ~315 °C. Ln-doping has always an inhibitory effect, shifting the conversion curves to higher temperatures. A similar detrimental effect has been obtained upon increasing Sm content on Cu/Ce_1-x_Sm_x_O_δ_ composites [26]. Ce-Pr and Ce-Nd are able to achieve full conversion at ~400 °C, whereas temperatures higher than 400 °C are required for Ce-La, Ce-Sm and Ce-Gd samples.

Addition of Cu (20 wt. %) to Ce-Ln mixed oxides improves the EtOAc conversion performance in all cases (Figure 8b). Cu/CeO_2_ and Cu/Ce-Pr samples demonstrated the best performance, with complete conversions of EtOAc at 280–290 °C. In relation to the catalytic efficiency of the remaining samples, the following order was found: Cu/Ce-Pr > Cu/Ce-Sm > Cu/Ce-Gd > Cu/Ce-Nd > Cu/Ce-La.

**Figure 9:**
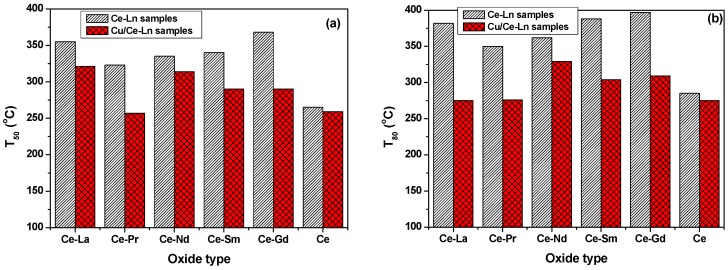
Comparison of EtOAc performance of CeO_2_ and Ce-Ln materials, with and without Cu (Ln: Gd, La, Pr, Nd, Sm), in terms of temperature required for 50% (**a**) and 80% (**b**) conversion (T_50_ and T_80_, respectively). Reaction conditions: [VOC]_in_ = 1000 mg_Carbon_/m^3^, GHSV = 60,000 h^−^^1^. The error for catalytic experiments is ±2 °C.

The effect of Cu addition to bare CeO_2_ or to Ce-Ln is more clearly depicted in Figure 9, which compares the temperatures required for 50% (a) and 80% (b) EtOAc conversion (T_50_ and T_80_, respectively) as a function of catalysts composition. T_80_ was chosen since not all oxide samples are able to completely convert EtOAc within the temperature range investigated. Loading with Cu has a positive effect on EtOAc conversion in all cases, lowering both the T_50_ and T_80_ temperatures. However, this effect is strongly dependent of the support. 

For bare CeO_2_ carrier, Cu does not have any important effect, revealing the superiority of ceria by itself. However, significant enhancements can be obtained by Cu addition to the mixed Ce-Ln oxides, in terms of lowering the T_50_ temperature by up to 80 °C (in the case of Ce-Gd) and T_80_ by up to 110 °C (for Ce-La). The impact of Ln- and/or Cu-doping on the VOCs oxidation performance is further discussed below.

## 3. Discussion

**Figure 10:**
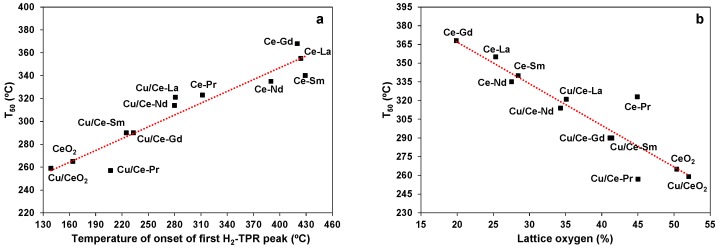
Correlation of 50% conversion temperature (T_50_) with: (**a**) the onset temperature of the first H_2_-TPR peak and (**b**) the amount of lattice oxygen for Ce-Ln and Cu/Ce-Ln samples (Ln: Gd, La, Pr, Nd, Sm). The error for catalytic experiments is ±2 °C, for TPRs ±5 °C and for lattice oxygen amount ±2%.

The present results clearly reveal that both Ln-doping (Ce-Ln samples) and Cu incorporation to mixed oxides (Cu/Ce-Ln samples) can affect the EtOAc oxidation (Figure 8 and Figure 9), but to a notably different extent. In general, Ln-doping has a detrimental effect on the catalytic performance, whereas Cu addition to bare CeO_2_ or Ce-Ln mixed oxides has a positive effect, mainly in the latter cases. These findings can be explained by taking into account the impact of Ln- and/or Cu-doping on the surface and redox properties of the mixed oxides. More specifically, the observed trend in catalytic activity (Figure 8 and Figure 9) is related to the reducibility sequence (Figure 2), showing that there is a close correlation between the redox behaviour of the materials and their EtOAc oxidation activity. That is shown in Figure 10, which depicts the relationship between T_50_ and onset temperature of the first H_2_-TPR peak (Table 1). A significant correlation is obtained, revealing the important role of catalyst reducibility on the activity towards oxidation of EtOAc. Moreover, a strong correlation was obtained between the catalytic performance (T_50_) and the relative amount of lattice oxygen (Figure 10b), *i.e.*, the O_I_ peak (%) determined by XPS (Table 2).

The observed correlations are in perfect agreement with the reaction mechanism proposed by Menon *et al.* [88] for the total oxidation of VOCs over Cu-based mixed oxides. It was revealed by temporal analysis of products (TAP) and isotopic labelling that the catalytic activity is mainly determined by weakly bound surface lattice oxygen atoms; Cu^2+^ species in close proximity with lattice oxygen species are considered responsible for VOC adsorption and consequent activation. In a similar manner, it was recently shown that cobalt incorporation into the ceria lattice notably enhanced the EtOAc oxidation performance, due to the increase of lattice oxygen ions; the replacement of Ce^4+^ by cobalt cations results in the change of lattice oxygen species concentration, reflected on the enhanced EtOAc degradation performance [89].

The present findings clearly reveal that EtOAc oxidation over transition metal oxides proceeds via a Mars-van Krevelen type redox mechanism, in agreement with relevant literature studies [16,28,29,35,40,46,57]. It involves the VOC activation, insertion of lattice oxygen species, and their subsequent replenishment by oxygen atoms originating from dioxygen in the gas phase [15,16,29,57]. In this mechanistic sequence, the abundance of weakly bound (easily reduced) surface lattice oxygen species is of major importance towards the facile oxidation of VOCs, demonstrating the key role of reducibility and lattice oxygen species on VOCs catalytic abatement.

It is also worth mentioning that the Cu/Ce-Pr sample, which is amongst the most effective catalysts, has a very low surface area (13 m^2^/g), implying that there is no particular correlation between the VOC oxidation performance and textural characteristics of the present samples. Moreover, both Cu/Ce-La and Cu/Ce-Nd show inferior performance (Figure 8b), in spite of their higher surface areas (Table 1). Hence it can be concluded that the redox/surface properties play a more important role than the textural characteristics in VOCs oxidation.

## 4. Materials and Methods

### 4.1. Material Synthesis

Five different Ce-containing oxides, *i.e.*, Ce_0.5_Gd_0.5_O_1.75_ (Ce-Gd), Ce_0.5_La_0.5_O_1.75_ (Ce-La), Ce_0.5_Pr_0.5_O_1.75_ (Ce-Pr), Ce_0.5_Nd_0.5_O_1.75_ (Ce-Nd) and Ce_0.5_Sm_0.5_O_1.75_ (Ce-Sm) were used as supports. Mixed oxides were synthesized by the wet impregnation method, using metal nitrates (>99.5 purity, Sigma Aldrich, Taufkirchen, Germany) as precursor salts. Initially, the desired amount of precursor salts was diluted in double distilled water, followed by heating at 80 °C until water evaporation. The resulting suspensions were dried overnight at 100 °C, followed by calcination at 550 °C for 2 h. Calcined mixed Ce oxides were then impregnated with an aqueous solution of Cu(NO_3_)_2_·3H_2_O to obtain Cu samples with 20 wt. % Cu nominal loading. The resulting samples were finally dried overnight at 100 °C and calcined at 550 °C for 2 h.

### 4.2. Characterization Studies

#### 4.2.1. Textural Characterization

The surface area of as prepared samples was determined by N_2_ adsorption isotherms at −196 °C, using the multipoint BET analysis method, in a Tristar Micromeritics 3000 flow apparatus (Micromeritics, Norcross, GA, USA). BET surface area was obtained according to Brunauer-Emmett-Teller (BET) method. The total pore volume was calculated based on the adsorbed nitrogen at the relative pressure of 0.99. Prior to the measurements, the samples were degassed at 250 °C overnight.

#### 4.2.2. XRD

The crystalline structure of the catalysts was determined by X-ray powder diffraction (XRD) in a PAN’alytical X’Pert MPD (PANalytical B.V., Almelo, The Netherlands) equipped with a X’Celerator detector (PANalytical B.V., Almelo, The Netherlands) and a secondary monochromator (Cu Kα = 0.15418 nm, 40 kV, 30 mA). The collected patterns were analysed qualitatively by HighScore Plus software and quantitatively by Rietveld refinement using PowderCell software, allowing the determination of crystallite sizes using Williamson-Hall model.

#### 4.2.3. TPR

Temperature programmed reduction (TPR) experiments were performed in a fully automated AMI-200 Catalyst Characterization Instrument (Altamira Instruments, Pittsburgh, PA, USA). In a typical TPR experiment, the sample (~50 mg) was placed in a U-shaped quartz tube located inside an electrical furnace and heated to 1000 °C at 10 °C/min heating rate under He flow rate of 29 cm^3^/min and H_2_ flow rate of 1.5 cm^3^/min.

#### 4.2.4. SEM

The surface analysis for morphological characterisation was carried out by scanning electron microscopy (SEM), using a FEI Quanta 400 FEG ESEM (15 keV) electron microscope (FEI Europe, Eindhoven, The Netherlands), The sample powders were mounted on double sided adhesive tape and observed at different magnifications under two different detection modes: secondary and backscattered electrons. Energy-dispersive X-ray spectroscopy (EDS) confirmed the nature of the components.

#### 4.2.5. TEM

Samples were also analysed by transmission electron microscopy (TEM), using a LEO 906E apparatus (Zeiss, Germany) operating at 120 kV (point resolution of 0.33 nm) equipped with a 4 Mpixel, CCD 28 mm × 28 mm digital camera from TRS (Zeiss, Germany). The samples were dispersed in ethanol in an ultrasonic bath, and collected on a formvar/carbon grid from Agar (Stansted, Essex, UK), dipped into the dispersion.

#### 4.2.6. XPS

The surface composition of the samples was determined by X-ray photoelectron spectroscopy (XPS) using a Kratos Axis Ultra HSA (Kratos Analytical Limited, Manchester, UK), with VISION software for data acquisition and CASAXPS software for data analysis. The effect of the electric charge was corrected by the reference of the carbon peak (284.6 eV). There is partial superimposing of C 1s with Ce 4s for all samples (and additional superimposing of C 1s with Gd 4p_1/2_ for Gd containing materials), thus additional care was taken with this correction.

### 4.3. Catalytic Activity Measurements

The catalytic evaluation was performed in a U-shaped quartz tube fixed-bed reactor with 6 mm internal diameter, placed inside a temperature controlled electrical furnace. A total air flow rate of 500 cm^3^/min, corresponding to a space velocity of 60,000 h^−1^, with an EtOAc composition of 1000 mg_Carbon_/m^3^ (~466.7 ppmV) was used. 50 mg of catalyst were mixed with inert SiC (carborundum, 0.2–0.5 mm) resulting in a total bed volume of *ca.* 0.5 cm^3^. Before oxidation experiments, the catalyst was pre-treated in air until 400 °C for 1 h. The catalyst was heated at a rate of 2.5 °C·min^−1^ until 400 °C under reaction conditions. The catalytic performance was evaluated by monitoring the EtOAc outlet concentration during temperature decrease from 400 °C to room temperature. The concentration of EtOAc in the effluent stream was measured by gas chromatography using a Master GC Dani (DANI Instruments S.p.A, Cologno Monzese, Italy) equipped with a FID detector. A silica-based CO_2_ non-dispersive infrared (NDIR) analyzer (Vaisala GMP222, Vaisala, Vantaa, Finland) was employed to continuously monitor the produced CO_2_.

## 5. Conclusions

In the present study the impact of lanthanide (Ln: Gd, La, Pr, Nd, Sm)-doping and/or copper incorporation to EtOAc oxidation performance of Ce_0.5_Ln_0.5_O_1.75_ and Cu/Ce_0.5_Ln_0.5_O_1.75_ catalysts was investigated. The following activity order for the Cu-free samples, in terms of EtOAc conversion, was found: CeO_2_ ≈ Ce_0.5_Pr_0.5_O_1.75_ > Ce_0.5_Sm_0.5_O_1.75_ > Ce_0.5_Gd_0.5_O_1.75_ > Ce_0.5_Nd_0.5_O_1.75_ > Ce_0.5_La_0.5_O_1.75_, showing the detrimental effect of lanthanide-doping. Cu incorporation to Ce-Ln oxides enhanced the catalytic performance without affecting the activity order. The best performance was obtained for Cu/CeO_2_ and Cu/Ce_0.5_Pr_0.5_O_1.75_, both samples showing complete conversion of EtOAc at ~290 °C. Strong correlations between the reducibility of the materials and the lattice oxygen availability with the EtOAc oxidation activity were found, highlighting the key role of these parameters on the catalytic performance. A Mars-van Krevelen type redox mechanism was considered, involving the participation of weakly bound/easily reduced lattice oxygen species, and their subsequent replenishment by gas phase oxygen. Therefore, the fine-tuning of noble metal-free mixed oxides towards the increase of surface oxygen mobility could lead to the development of low-cost and highly-efficient VOCs catalysts.

## Figures and Tables

**Table 1:** Main physicochemical characteristics of Ce-Ln and Cu/Ce-Ln materials (Ln: Gd, La, Pr, Nd, Sm) obtained by BET, XRD and TPR analyses. Onset temperature of H_2_-TPR peaks is shown in bold, peak maxima in plain text and peak shoulders in italic.

Samples	BET Analysis		XRD Analysis		TPR Analysis	
	S_BET_ (m^2^/g)	Total Pore Volume (cm^3^/g)	Phase Detected (%*v*/*v*) ^b^	Crystallite Size (nm)	Lattice Parameter (Å) ^c^	Peak Temperatures (°C) ^a^
CeO_2_	72	0.27	CeO_2_ cerianite	15.9 ± 0.5	5.41	**164**, 507, 780
Cu/CeO_2_	45	0.15	CeO_2_ cerianite (55.2%)	13.2 ± 0.2	5.41	**139**, 180, *220*, 232, 836
			CuO tenorite (44.8%)	70 ± 1		
Ce-Gd	5	0.09	CeO_2_ cerianite (81.7%)	16 ± 1	5.43	**420**, 654
			Gd_1-x_Ce_x_O_y_ cubic (18.3%)	31 ± 5	5.62	
Cu/Ce-Gd	3	0.05	CeO_2_ cerianite (48.8%)	25 ± 2	5.42	**233**, 293, 310, 402
			Gd_1-x_Ce_x_O_y_ cubic (21.9%)	51 ± 5	5.47	
			CuO tenorite (29.3%)	10 ± 1		
Ce-La	25	0.14	CeO_2_ cerianite (35.2%)	18 ± 1	5.43	**424**, 551, 644
			La_1-x_Ce_x_O_y_ (64.8%)	34 ± 1	5.56	
Cu/Ce-La	23	0.08	La_1-x_Ce_x_O_y_ (66.2%)	23 ± 2	5.51	**281**, *326*, 343, 364
			La_2_CuO_4_ cubic (8.6%)	49 ± 7	5.36	
			CuO tenorite (25.2%)	11 ± 2		
Ce-Pr	22	0.15	Pr_1-x_Ce_x_O_y_	13 ± 1	5.43	**312**, 457, *484*, 566
Cu/Ce-Pr	13	0.08	CeO_2_ cerianite (87.8%)	19 ± 1	5.41	**207**, *308*, 328, *347*
			CuO tenorite (12.2%)	48 ± 5		
Ce-Nd	35	0.18	Nd_1-x_ Ce_x_O_y_ *	9 ± 1	5.49	**390**, 551, 810
Cu/Ce-Nd	33	0.08	Nd_1-x_ Ce_x_O_y_ (62.7%)	11 ± 1	5.47	**280**, *325*, 358, *370*
			Nd_4_Cu_2_O_7_ (7.9%)	52 ± 5		
			CuO tenorite (29.4%)	11 ± 1		
Ce-Sm	21	0.17	CeO_2_ cerianite (77.8%)	18 ± 1	5.44	**429**, 598
			Sm_1-x_Ce_x_O_y_ (22.2%)	20 ± 5	5.64	
Cu/Ce-Sm	19	0.08	CeO_2_ cerianite (53.3%)	15 ± 1	5.45	**255**, *295*, 326, *348*
			Sm_2_CuO_4_ tetragonal (9.2%)	39 ± 5		
			Sm_1-x_Ce_x_O_y_ cubic x ≈ 0.5 (7.5%)	5 ± 1	5.65	
			CuO tenorite (30.0%)	12 ± 1		

^a^ The error for TPR experiments is ±5 °C; ^b^ Obtained by Rietveld analysis; ^c^ Calculated only for cubic structures.; * The Rietveld analysis also converges to a solution with 47.5% (*v*/*v*) of CeO_2_ (a = 5.45 Å) and 52.5% (*v*/*v*) of Nd_1-x_Ce_x_O_y_ (a = 5.54 Å).

**Table 2:** Surface characteristics (determined by XPS) of Ce-Ln and Cu/Ce-Ln (Ln: Gd, La, Pr, Nd, Sm) materials.

Samples	XPS Atomic Ratios ^a^			Relative Concentrations (%) ^c^			
	Cu/Ce + Ln (0.70) ^b^	Ce/Ce + Ln (0.50) ^b^	Ln/Ce + Ln (0.50) ^b^	Cu^+^	Ce^3+^	O_I_	O_I_/O_II_
CeO_2_	-	1	-	-	17.82	50.37	1.01
Cu/CeO_2_	0.62	1	-	13.20	18.79	52.02	0.90
Ce-Gd	-	0.04	0.96	-	23.13	19.89	0.25
Cu/Ce-Gd	0.08	0.02	0.98	6.41	18.27	41.14	0.41
Ce-La	-	0.43	0.57	-	20.12	25.34	0.34
Cu/Ce-La	0.29	0.40	0.60	<1	23.53	35.10	0.54
Ce-Pr	-	0.40	0.60	-	20.06	44.93	0.92
Cu/Ce-Pr	0.94	0.59	0.41	^d^	20.60	45.01	0.82
Ce-Nd	-	0.21	0.79	-	20.92	27.53	0.37
Cu/Ce-Nd	0.17	0.26	0.74	29.20	22.26	34.30	0.49
Ce-Sm	-	0.34	0.66	-	23.20	28.46	0.40
Cu/Ce-Sm	0.69	0.31	0.69	<1	18.38	41.39	0.63

^a^ estimated from XPS spectra; ^b^ calculated from the nominal catalyst composition of 20 wt. % Cu/Ce_0.5_Ln_0.5_O_1.75_; ^c^ estimated from the integrated areas of the respective XPS peaks; ^d^ superimposition with the Pr 3d peak.
